# Editorial: Genomics and Effectomics of Filamentous Plant Pathogens

**DOI:** 10.3389/fgene.2021.648690

**Published:** 2021-02-03

**Authors:** Wenwu Ye, Qinhu Wang, Sucheta Tripathy, Meixiang Zhang, Ramesh R. Vetukuri

**Affiliations:** ^1^The Key Laboratory of Plant Immunity/Department of Plant Pathology, Nanjing Agricultural University, Nanjing, China; ^2^State Key Laboratory of Crop Stress Biology for Arid Areas, College of Plant Protection, Northwest A&F University, Xianyang, China; ^3^Structural Biology & Bioinformatics Division, CSIR - Indian Institute of Chemical Biology, Kolkata, India; ^4^Department of Plant Breeding, Swedish University of Agricultural Sciences, Alnarp, Sweden

**Keywords:** oomycete, fungi, genomics, effector, small RNAs, plant-pathogen interactions

Fungi and oomycetes are filamentous microorganisms that include a diversity of highly developed pathogens of plants. Notorious plant diseases, such as rice blast (*Magnaporthe oryzae*), wheat head blight and crown rot (*Fusarium graminearum* and *F. pseudograminearum*), wheat rust (*Puccinia* spp.), Botrytis bunch rot (*Botrytis cinerea*), and pear or apple canker (*Valsa pyri*) are caused by fungal pathogens, while soybean Phytophthora root rot and Pythium damping-off (*Phytophthora sojae* and *Pythium* spp.), potato late blight (*Phytophthora infestans*), pepper blight (*Phytophthora capsici*), and fruit and vegetable downy mildew (*Pseudoperonospora* and other Peronosporaceae spp.) are caused by oomycete pathogens. Over the past 15 years, the genomes of more than 150 plant pathogenic fungi and oomycetes have been sequenced. The fast-accumulating “omic” data and bioinformatics techniques have significantly promoted the understanding of molecular mechanisms for plant-pathogen interactions.

Accumulating evidence has revealed that a co-evolutionary arms race with host plants has shaped the genomes of the filamentous plant pathogens into diverse architectures, in addition to producing some common features in phylogenetically unrelated species. Typically, the pathogens have evolved a large repertoire of secreted virulence proteins, called effectors, which are able to target different compartments of host cells to facilitate colonization and infection. Some effectors can be recognized by the plant immune system and trigger immunity, while others may escape recognition of the host by rapid evolution. This Research Topic aims to showcase the utility of genomics and effectomics to gain insights into the pathogenesis and virulence factor evolution of filamentous plant pathogens by revealing the breadth of novelty that was in the past largely untapped. It finally comprises 19 submissions from experts in the field and covers a broad range of themes. We have broadly grouped the articles under the following ten themes:

## Genome Assembly

Long-read sequencing can greatly improve the genome assembly quality, thus is also helpful for genome annotation. Wu et al. generated the first long-read sequencing-based *de novo* genome assembly for the wheat leaf rust pathogen *Puccinia triticina* (Pt) strain Pt104. Compared to the previous Pt race1 assembly, the Pt104 assembly reduces the contig number by 91-fold and improves the contig N_50_ by 4-fold. Comparative genomics among Pt104 and six additional strains, which have differential virulence profiles on different wheat varieties carrying leaf rust resistance gene *Lr26, Lr2a*, or *Lr3ka*, provided evidence for the identification of 31–38 candidates for each corresponding avirulence gene. Based on an updated genome assembly of *Phytophthora parasitica* using PacBio long sequence reads, Panabières et al. identified several families of tandemly repeated sequences varying in size, copy number, and sequence conservation. Two abundant families, *PpSat1* and *PpSat2*, displayed typical features of satellite DNA but differed by their length, sequence, organization, genomic environment, and evolutionary dynamics. Characterization of transcripts of the two families further suggested that these satellite DNA families likely play a conserved role within oomycete pathogens.

## Effector Identification

Identification of secretome and candidate effectors from phytopathogen genomes is a fundamental work for pathogenesis research. In the genome of *Pseudoperonospora humuli* isolate OR502AA, Purayannur et al. predicted 1,250 secretome-coding genes, and 296 RxLR or RxLR-like effector-encoding genes. Based on 12 different isolates collected from various hop cultivars, they further identified a set of core effectors that showed transcription evidence and elevated expression during infection. In *Phytophthora betacei*, Rojas-Estevez et al. found that the proteome has both significantly higher numbers of whole proteome (40,543) and predicted secretome (5,653), and the proportion of secretome in the proteome is also slightly higher (13.9%) than other *Phytophthora* spp. There is an extremely large repertoire of RxLR effectors (791), among which 203 RxLR effectors were specific in *P. betacei*. The avirulence RxLR effectors identified in other *Phytophthora* spp., such as *Avr1, Avr3b, Avr4*, and *Avrblb1*, were likely conserved in *P. betacei*.

## Effector Evolution

In many plant pathogens, some effectors can be detected by corresponding resistance proteins from their host plants and activate immunity. The high selection pressure from host resistance usually results in a high degree of variability of pathogens. Wang et al. investigated the distribution, variation, and evolution of the corresponding *Avr* gene *AvrPiz-t* among 312 *M. oryzae* isolates collected from Yunnan rice production areas of China. The data revealed that *AvrPiz-t* evolved to virulent from avirulent forms via point mutation, retrotransposon, shift mutation, and structure variance under field conditions. In *P. sojae*, all previously identified *Avr* genes belong to the RxLR effector family. Zhang et al. investigated the genomic variation of 25 *P. sojae* isolates by high-throughput genome re-sequencing, and found that the *P. sojae* isolates possess varying numbers of RxLR effectors with diverse sequences. Forty two core RxLR effectors are assumed to be important for infection, and several novel variants of avirulent RxLR effectors leading to the evading of host resistance were identified.

## Effector Functional Screening

Many effectors exhibit specific transcriptional induction during early stages of pathogen infection. Zhao et al. performed RNA-seq and identified a set of upregulated genes upon *Puccinia triticina* infection, including 79 genes predicted as possible effectors. Among the effector candidates which contained a PNPi-like or a CFEM motif, four PNPi-like effector candidates showed physical interactions with wheat NPR1 protein in yeast two-hybrid assay. Transient expression of one CFEM effector candidate in *Nicotiana benthamiana* accelerated the progress of cell death and promoted the accumulation of reactive oxygen species.

## Apoplastic Cell Death-Inducing Effectors

Li et al. reviewed the latest advances in the identification of apoplastic cell death-inducing proteins (CDIPs) from plant pathogenic oomycetes and fungi, and discussed the role of many apoplastic CDIPs as essential virulence factors. At the same time, apoplastic CDIPs have been documented to be recognized by plant cells as pathogen-associated molecular patterns (PAMPs). The recent findings of extracellular recognition of apoplastic CDIPs by plant leucine-rich repeat-receptor-like kinases (RLKs) or receptor-like proteins (RLPs) have greatly advanced our understanding on how plants detect microbial patterns and mount a defense response.

## Transcription Factors

Transcription factor (TF) is a class of sequence-specific DNA-binding factors playing important roles in the development and pathogenicity of plant pathogens. Kange et al. identified VpFSTF_1_, a fungal-specific TF from the pear canker pathogen *Valsa pyri*. The gene knockout mutants lost the ability to form fruiting bodies along with the reduced virulence, and were sensitive to increasing concentrations of hydrogen peroxide and salicylic acid. RNA-seq analysis revealed 69 candidate downregulated genes related to virulence, and five promoters proposed to be directly or indirectly targeted by VpFSTF1. Wang et al. compared the transcriptomes of gene-silenced and wild-type strains and identified candidate downstream genes regulated by a histone deacetylase in *Phytophthora infestans*. Among 18 candidate genes related to α hormones biosynthesis, overexpression of a gene encoding the NF-Y TF increased the production of hormone α2. Yin et al. report that the plant-specific RWP-RK TF family is also widely present in the Stramenopila kingdom, particularly among the oomycetes, with 12–15 members per species. In addition to protein sequences and DNA-binding domains, the transcriptional activities of orthologous RWP-RK genes in oomycetes were also conserved, and some genes may be associated with pathogenicity.

## Pentatricopeptide Repeat Proteins

Pentatricopeptide repeat (PPR) proteins are a large family of modular RNA-binding proteins that mediate several aspects of gene expression, but compared to plants, the function of PPR proteins is still largely unknown in filamentous plant pathogens. Wang et al. reported that *FpPPR1*, a PPR encoding gene, is essential for asexual development, sporulation, and pathogenesis in *F. pseudograminearum*. RNA-seq revealed significant transcriptional changes in the *Fpppr1* deletion mutant, and several differentially regulated genes may function in mating type, heterokaryon incompatibility, and dysfunction of mitochondria-mediated oxidative stress, and explain the functions of FpPPR1.

## Carbohydrate-Active Enzymes

Carbohydrate-active enzymes (CAZymes) of plant pathogens are involved in the degradation of the host cell wall and storage compounds, and many have been revealed as virulence factors. In *Phytophthora sojae*, Tan et al. identified PsGH7a, which encodes a glycoside hydrolase (GH)7 family cellobiohydrolase and was highly induced during the cyst germination and infection stages. The *PsGH7a* knockout mutants showed reduced virulence on susceptible soybean. Notably, PsGH7a protein triggers hypersensitive response in diverse plants, and it is highly conserved in oomycetes. de Vries et al. performed comparative genomics to understand how the repertoire of the carbohydrate esterase (CE)1 and CE10-encoding gene families is shaped across oomycete evolution. These genes are mainly induced in the analyzed oomycete plant pathogens and some homologous genes show lifestyle-specific gene expression levels during infection, with hemibiotrophs showing the highest expression levels. Liang et al. compared the full CAZyme complement among nine *Pythium* (two are mycoparasitic) and four *Phytophthora* species. They found that 20 CAZyme families involved in the degradation of cellulose, hemicellulose, glucan, and chitin were expanded in, or unique to, mycoparasitic *Pythium* species. Three families might be expanded via tandem gene duplication, and five families were likely via horizontal gene transfer.

## Post-Translational Modification

Protein lysine 2-hydroxyisobutyrylation (K_hib_) and histone lysine lactylation (Kla) are two newly discovered post-translational modification (PTM). Xu et al. present research on a proteome-wide analysis of K_hib_ protein in *Botrytis cinerea*. A total of 5,398 K_hib_ sites from 1,181 proteins were identified. Functional annotations showed that the K_hib_ proteins are widely distributed in cellular compartments and involved in diverse cellular processes, and significantly, 37 K_hib_ proteins were proposed to function in different aspects of pathogenicity regulation. Gao et al. describe a global lysine lactylome analysis in *B. cinerea*. Among the 166 proteins with 273 Kla sites identified, 88% were predicted to distribute in nucleus, mitochondria, and cytoplasm, and 12 may participate in fungal pathogenicity. The combined datasets of K_hib_ and Kla in *B. cinerea* provide a good foundation for further explorations of PTM in plant fungal pathogens.

## Pathogen-Responsive Plant Small RNAs

Small RNAs are critical for plant immunity against diverse virus, bacteria, and a couple of eukaryotic pathogens. Based on a high-throughput sequencing approach, Zhu et al. identify 293 known and six novel small RNAs (miRNAs or siRNAs) in *Arabidopsis thaliana*, which were responsive to *Phytophthora capsici* at early stage of infection. The predicted target genes of 33 selected miRNAs were enriched in pathways of starch and sugar metabolism, spliceosome, and plant-pathogen interaction, indicating that the splicing machinery and pathogenesis-related proteins play important roles in response to *P. capsici* infection. Based on a powdery mildew (PM)-susceptible cucumber line and a PM-resistant line, Xu et al. applied small RNA and degradome sequencing to identify PM-responsive miRNAs and their target genes. The comparative study highlighted an extensive genotype-specific response to PM infection that was different from the common responses in the resistant and susceptible genotypes. Four miRNAs and their target genes were found to play critical roles in the PM-inoculated cucumber leaves.

Overall, this Research Topic showcases a broad range of articles which illustrate the utility of multi-omics approaches to investigate genomic (and effectomic) features associated with the development, pathogenicity, and virulence evolution of filamentous plant pathogens. Future advances in genomic technology will undoubtedly reveal further novelty and diversity of pathogenic mechanisms for a better understanding of plant-pathogen interaction.

## Author Contributions

WY, QW, ST, MZ, and RV co-edited the Research Topic. WY wrote the editorial. QW, ST, MZ, and RV edited and approved the final version of the editorial. All authors contributed to the article and approved the submitted version.

## Conflict of Interest

The authors declare that the research was conducted in the absence of any commercial or financial relationships that could be construed as a potential conflict of interest.

